# Characterization of Vulnerable and Resilient Spanish Adolescents in Their Developmental Contexts

**DOI:** 10.3389/fpsyg.2016.00983

**Published:** 2016-07-04

**Authors:** Carmen Moreno, Irene García-Moya, Francisco Rivera, Pilar Ramos

**Affiliations:** ^1^Developmental and Educational Psychology, University of SevilleSevilla, Spain; ^2^Department of Behavioral Sciences, University of HuelvaHuelva, Spain

**Keywords:** adolescence, resilience, vulnerability, family functioning, global health score

## Abstract

Research on resilience and vulnerability can offer very valuable information for optimizing design and assessment of interventions and policies aimed at fostering adolescent health. This paper used the adversity level associated with family functioning and the positive adaptation level, as measured by means of a global health score, to distinguish four groups within a representative sample of Spanish adolescents aged 13–16 years: maladaptive, resilient, competent and vulnerable. The aforementioned groups were compared in a number of demographic, school context, peer context, lifestyles, psychological and socioeconomic variables, which can facilitate or inhibit positive adaptation in each context. In addition, the degree to which each factor tended to associate with resilience and vulnerability was examined. The majority of the factors operated by increasing the likelihood of good adaptation in resilient adolescents and diminishing it in vulnerable ones. Overall, more similarities than differences were found in the factors contributing to explaining resilience or vulnerability. However, results also revealed some differential aspects: psychological variables showed a larger explicative capacity in vulnerable adolescents, whereas factors related to school and peer contexts, especially the second, showed a stronger association with resilience. In addition, perceived family wealth, satisfaction with friendships and breakfast frequency only made a significant contribution to the explanation of resilience. The current study provides a highly useful characterization of resilience and vulnerability phenomena in adolescence.

## Introduction

Fostering wellbeing is one of the current priorities of international agendas in health promotion (WHO, 2012, 2014), and adolescence has been considered to be a key developmental stage for this objective (WHO, 2014). Scientific evidence on factors that help mitigate risk or promote good adjustment despite adversity is crucial to governments and international agencies, which need to efficiently and effectively invest their resources. Positive and negative factors for wellbeing accumulate throughout life and health promotion interventions, which maximize protective factors while minimizing risks, can be successful in achieving wellbeing gains (Marmot, 2010). Resilience research, which analyses risk and protective factors to understand positive development under adverse circumstances, therefore presents itself as a particularly valuable approach that can provide the foundations for the design of effective health promotion and preventive interventions (Roosa, 2000).

More specifically, the value of resilience studies for the design and evaluation of health promotion interventions is apparent for the following reasons. First, resilience research provides critical information about key factors that help reduce potential harm and encourage positive adaptation (Masten, 2014). Each identified protective or vulnerability factor offers a possible focus of intervention (Olsson et al., 2003). Furthermore, the advantage of these studies is that they not only provide a list of intervention targets, but also emphasize the most relevant factors for different population groups and adversity levels (Luthar and Cicchetti, 2000).

Additionally, in highlighting an individual's positive adaptation resilience studies facilitate a change of approach (Luthar and Cicchetti, 2000; Olsson et al., 2003; Fergus and Zimmerman, 2005). Thus, resilience is in line with the perspective shift which has gradually taken place in different disciplines, including psychology, in the last decades: from the reduction of existing problems and exclusive emphasis on deficit and risk, to a focus on the development and promotion of health resources and assets (Morgan et al., 2010).

Lastly, it is important to bear in mind that the utility of resilience research goes further than merely understanding the processes linked to adversity. According to existing evidence, protective factors (as vulnerability ones) are not specific to situations of adversity, but they are the manifestation of basic adaptational systems that come into play in a variety of situations (Masten and Coatsworth, 1998; Masten, 2001). Therefore, increasing our knowledge about resilience and vulnerability phenomena provides useful evidence for intervention and evaluation in adversity contexts and helps to better understand and promote positive development in the general population.

In order for scientific research to make a significant contribution to the design and evaluation of interventions and policies, it is fundamental that studies on resilience (as well as those on vulnerability) include a clear definition and operationalization of the terminology involved (Luthar and Cicchetti, 2000; Masten, 2014; Luthar et al., 2015). In this regard, resilience is defined as “a dynamic process encompassing positive adaptation within the context of significant adversity” (Luthar et al., 2000, p. 543). There is a wide consensus that the two criteria implicit in this definition must be met in order to identify resilience. Indeed, exposure to adversity and some evidence of positive adaptation have been referred to as the two “judgements,” “dimensions,” “sides” or “coexisting conditions” of resilience (Masten and Coatsworth, 1998; Luthar et al., 2000, 2015; Luthar and Cicchetti, 2000; Masten, 2001; Rutter, 2006).

The adversity element has been defined by characteristics as diverse as: an experience of war or catastrophe (Masten and Narayan, 2012), low economic status (Buckner et al., 2003), belonging to minority groups (Sandín-Esteban and Sánchez-Martín, 2015), living in disadvantaged neighborhoods (Tiêt and Huizinga, 2002) and an individual's or caregiver's disorders or illnesses (Werner and Smith, 1982). Nevertheless, the key defining characteristic of adversity is that a significant threat to development or demonstrable risk must be present (Luthar and Cicchetti, 2000; Masten, 2001). More specifically, adversity is defined by “current or past hazards judged to have the potential to derail normative development” (Masten, 2001, p. 228) and it “typically encompasses negative life circumstances that are known to be statistically associated with adjustment difficulties” (Luthar and Cicchetti, 2000, p. 858).

In this regard, putting key adaptational systems in danger, including the relationship with loving and competent adult caregivers in a family context, is amongst the principal hazards to human development (Masten, 2001). Extant evidence has documented the fundamental links between the quality of parent-child relationships and adolescent development and adjustment (Steinberg and Silk, 2002; Clarke-Stewart and Dunn, 2006). In this sense, family context has a very strong influence on the person from the beginning of life and through multiple channels. No wonder, therefore, that family is the center of many adaptation and human development studies in this field (Masten and Shaffer, 2006). Hence, low-quality parent-child relationships (García-Moya et al., 2013b) or the existence of problems in the family (Fergusson and Linskey, 1996) have been considered to be key elements in defining an adverse situation. Accordingly, low scores in a composite factorial measure of the quality of parent-child relationships (García-Moya et al., 2013a) will be used as the indicator of adversity in the present study.

In defining positive adaptation, resilience research is especially varied. Luthar et al. (2000, 2015) concluded that a single criterion to establish the best adaptation indicator for any given study does not exist. External criteria such as behavioral adjustment and social competence have tended to predominate (Olsson et al., 2003) but internal criteria including emotional health, life satisfaction or absence of emotional distress are increasingly seen as similarly important indicators of positive adaptation (Masten and Reed, 2005). Furthermore, some revealing studies show that individuals showing positive adaptation according to external competence criteria can still experience internalizing symptoms and health problems (e.g., Luthar et al., 1993). Drawing on this evidence, we selected a global health score, which encompasses self-rated health, psychosomatic complaints, health-related quality of life and life satisfaction, as the indicator of positive adaptation in the present study. This is not to say that positive adaptation is synonymous to health or wellbeing, but we made the conceptually-informed decision to give priority to the aforementioned internal dimensions of health to define positive adaptation. More specifically, the global health score (Ramos et al., 2010) was selected because of its relevance for the kind of adversity examined (Karademas et al., 2008; Jiménez-Iglesias et al., 2015), as well as being a sound composite factorial score that encompasses multiple domains of health and has shown good psychometric properties in adolescents (Ramos et al., 2012). Specifically, using the global health score as the criterion for positive adaptation fits with one of the approaches mentioned in a seminal chapter about measurement issues in the empirical study of resilience, underlining that the assessment of positive adaptation “must be tied in to the particular risk domain being studied” and “rests on multiple-item instruments, typically with well-documented psychometric properties, that provide assessments on the continuum between adjustment and maladjustment” (Luthar and Cushing, 1999, pp. 139–140). Furthering the definition of the constructs related to resilience and adaptation, some authors (Tiêt and Huizinga, 2002) have proposed an interesting classification of individuals based on their level of exposure to adversity and the resulting adaptation shown, which divides the population into four large groups. Two of the groups show expected results in accordance with their level of exposure to adversity: low-risk—good adaptation (*competent* or *unchallenged*) and high-risk—bad adaptation (*maladaptive*). The paradox occurs in the remaining two groups: those that are exposed to high-risk but show good adaptation and those that, despite being exposed to low levels of risk, exhibit low competence levels. The first of these latter two groups constitutes the sample of interest in resilience studies whereas the second group, although rarely studied, could offer interesting information about vulnerability factors in the normative population.

After establishing the group or groups of interest, the next step is to identify which factors facilitate (protective factors) or inhibit (vulnerability factors) positive adaptation in the given context. Research has tended to classify these factors using a theoretical framework which distinguishes three fundamental levels: individual-level, family-level, and extrafamily-level factors (Masten and Coatsworth, 1998; Luthar and Cicchetti, 2000; Olsson et al., 2003).

On the individual level, self-esteem, self-efficacy, and intellectual capacity have been extensively studied in classic literature on resilience as determinant factors on the individual level (Masten and Coatsworth, 1998; Dumont and Provost, 1999; Hamill, 2003). Nevertheless, the claim that positive self-perception along with confidence in one's efficacy and motivation to engage in the environment are fundamental for successful adaptation (Masten, 2001) justifies the need to explore the role of other constructs with clear links to the aforementioned description. Regarding positive self-perception, satisfaction with body image is one aspect that has been considered especially influential in adolescence (Tiggemann, 2005). Confidence in one's efficacy and motivation to engage in the environment are linked to some novel constructs in positive psychology that are likely to play a significant role in explaining positive adaptation, such as sense of coherence (Antonovsky, 1987) and curiosity and exploration (Kashdan et al., 2009). Finally, another fundamental factor is emotional regulation. This skill, which is closely related to intellectual functioning, is currently receiving special scientific attention since it seems to be fundamental for successful coping and good behavioral, emotional and social adjustment (Lengua, 2002; Buckner et al., 2003). The present study will try to further the understanding of individual-level factors by exploring the aforementioned constructs that, despite having connections with well-known individual factors in resilience studies, have not usually been included in previous resilience research.

Along with them, we will analyse the role of lifestyles that, despite their significant contribution to health and wellbeing, have also received little attention to date (Elliot, 1993; Ramos, 2010). Regarding tobacco, alcohol and cannabis use, the absence of these risk behaviors has been predominantly used as criteria for defining adaptation (for a review, see Fergus and Zimmerman, 2005) or its presence has been analyzed as a risk indicator (Anteghini et al., 2001). The associations between resilience and healthy lifestyles, such as eating habits, dental hygiene and physical activity, has also been rarely explored in classic studies. Nonetheless, physical activity, for example, has been highlighted as a relevant factor when explaining resilience due to its protective effects on health in stress situations (Gerber and Pühse, 2009; Silverman and Deuster, 2014) or the fact that it tends to be incompatible with some health-threatening activities or risk behaviors, such as alcohol and other substances abuse (Pate et al., 1996). Consequently, examining the associations between lifestyles and resilience is of unquestionable interest.

On the family level, besides aspects related to the aforementioned quality of relationships and processes in the family context (which will be used to define adversity in the present study), it is worth exploring the contribution of the families' socioeconomic status (Masten and Coatsworth, 1998). A good socioeconomic position is associated with access to material, cultural and educational resources, making it a significant source of social capital (Bornstein and Bradley, 2003), whereas low family affluence limits access to the aforesaid resources and could become a significant source of stress, having negative consequences on children's development (Conger et al., 2000). Unlike objective indicators, subjective measures of socioeconomic status have not generally been analyzed in resilience studies. However, the study of socioeconomic inequalities in health indicates that subjective perceptions of wealth have a strong predictive capacity regarding adolescent health (Goodman et al., 2001) and their significant effects on health remain even after controlling for objective measures such as educational level, parents' occupation and family affluence (Elgar et al., 2016).

Lastly, on the extrafamily level, experiences of belonging and efficacy, such as a positive school climate and experiences of academic achievement, can significantly contribute to positive adaptation outcomes (Masten and Coatsworth, 1998), whereas bullying episodes can hamper them (McVie, 2014). Significant extrafamily relationships with important adults, including teachers (DuBois et al., 1992; Masten and Coatsworth, 1998), as well as the contribution of peer support and the degree in which peers provide positive or adjusted models of behavior (e.g., Jain et al., 2012) have also been emphasized. The present study will consider all the aforementioned aspects.

Therefore, the selection of variables in the present study is supported by an ample consensus on the need to analyse factors from individual, family and extrafamily levels in order to obtain a detailed view of the factors associated with resilience and vulnerability (Masten and Coatsworth, 1998; Luthar and Cicchetti, 2000; Olsson et al., 2003). In addition, the selection of variables is guided by an explicit effort to explore relevant content from those levels that have not been sufficiently examined in resilience research so far. Thus, the present study will try to further the understanding of individual-level factors by exploring emotional regulation along with other constructs such as satisfaction with body image, sense of coherence and curiosity and exploration that, despite having connections with well-known individual factors in resilience studies, have not usually been included in previous resilience research. Similarly, because lifestyles contribute significantly to wellbeing, the selection of variables included breakfast frequency, physical activity and substance use, which have also received little attention in the study of resilience. On the family level, a similar rationale motivated the selection of perceived family wealth as the measure of socioeconomic status, instead of the objective indicators which have dominated previous resilience research. Finally, on the extrafamily level, the selected variables (including academic achievement, classmate and teacher support, bullying victimization, peer support, and models of behavior in the peer group) ensure simultaneous consideration of the most frequently mentioned factors on this level.

Accordingly, this paper starts by using the criteria on adversity and positive adaptation described above to identify two reference groups within a representative sample of adolescents: those that showed good global health despite having a low-quality family environment (*resilient*), and those that showed poor health even with high-quality parent-child relationships (*vulnerable*). Afterwards, drawing on the classification by Tiêt and Huizinga (2002), the phenomena of resilience and vulnerability are characterized by comparing them to groups of *maladaptive* (high risk, poor adaptation) and *competent* (low risk, good adaptation) adolescents, respectively.

The aim of the paper is to characterize resilience and vulnerability in adolescents, considering an ample number of potential protective and vulnerability factors that were selected from the three main levels described in scientific literature (individual, family, and extrafamily). The selection of the specific factors used in this study is also intended to initiate a new direction by exploring relevant constructs for positive adaptation in adolescence which had not received sufficient attention in classic resilience research, amongst others, satisfaction with body image, sense of coherence, curiosity and exploration, and diverse lifestyles.

In short, after conducting preliminary analyses on the differences among resilient, vulnerable, competent, and maladaptative adolescents in individual factors (including psychological variables and lifestyles), family socioeconomic status and extrafamily factors (including those from the school context and the peer context), the ability of those factors (as independent variables) to explain the dependent variables resilience (vs. maladaptation) and vulnerability (vs. competence) is examined. A detailed list of research question is presented in Table 1.

**Table 1:** **Research questions in the present study**.

**Research question 1** How do the four groups of adolescents analyzed in this paper (maladaptative, resilient, competent, and vulnerable) characterize and differentiate from each other in relation to the three sets of variables analyzed: individual factors (including psychological variables and lifestyles), family socioeconomic status and extrafamily factors (including those from the school context and the peer context)?
**Research question 2** Which factors (individual, familial, and extrafamiliar) are useful to understand adaptation in high adversity contexts? In other words: which factors (individual, familial, and extrafamilial) are useful to distinguish between resilient and maladaptative adolescents?
The following *specific questions* will be answered before addressing research question 2: 2a. Which psychological factors (sense of coherence, emotional regulation, curiosity and exploration, perceived body image and satisfaction with body image) distinguish between resilient and maladaptative adolescents? 2b. Which factors related to lifestyles (breakfast frequency, fruit consumption, physical activity, dental hygiene, tobacco, alcohol and cannabis use) distinguish between resilient and maladaptative adolescents? 2c. Which family socioeconomic factors (father's educational level, mother's educational level and perceived family wealth) distinguish between resilient and maladaptative adolescents? 2d. Which factors referring to school (perceived academic achievement, feelings toward school and perceived teacher support) distinguish between resilient and maladaptative adolescents? 2e. Which factors referring to peer group (perceived peer support, models of behavior, satisfaction with friendships, having been bullied and having bullied others) distinguish between resilient and maladaptative adolescents?
**Research question 3** Which factors (individual, familial, and extrafamilial) are useful to understand adaptation in low adversity contexts? In other words: which factors (individual, familial, and extrafamilial) are useful to distinguish between vulnerable and competent adolescents?
The following *specific questions* will be answered before addressing research question 3: 3a. Which psychological factors (sense of coherence, emotional regulation, curiosity and exploration, perceived body image and satisfaction with body image) distinguish between vulnerable and competent adolescents? 3b. Which factors related to lifestyles (breakfast frequency, fruit consumption, physical activity, dental hygiene, tobacco, alcohol, and cannabis use) distinguish between vulnerable and competent adolescents? 3c. Which family socioeconomic factors (father's educational level, mother's educational level and perceived family wealth) distinguish between vulnerable and competent adolescents? 3d. Which factors referring to school (perceived academic achievement, feelings toward school and perceived teacher support) distinguish between vulnerable and competent adolescents? 3e. Which factors referring to peer group (perceived peer support, models of behavior, satisfaction with friendships, having been bullied and having bullied others) distinguish between vulnerable and competent adolescents?

This approach is designed to identify important factors for adaptation in adverse and non-adverse contexts respectively, but it may also provide valuable findings that contribute to informing the debate on whether some factors contribute to positive development in the face of adversity but have little impact in the absence of it or whether there are some common protective and risk factors associated with positive adaptation irrespective of the level of adversity exposure (Roosa, 2000). Also, on the potential implications and contributions offered by the present study, it has been stated that although “this kind of epidemiological research does not unpack the processes by which each individual is impacted by contextual experience, it does document the multiple factors in the environment that are candidates for more specific analyses (Sameroff, 2010, p. 14).” The aforementioned factors and levels do not operate independently, rather they relate amongst themselves in people's lives (Fergus and Zimmerman, 2005). For this reason, approaches which provide an ample characterization of resilience and vulnerability phenomena while taking into account a significant number of the aforementioned factors (usually referred as person-focused approaches) provide a very valuable complementary approach (Masten, 2001).

## Method

### Participants

Data were obtained from the Health Behavior in School-aged Children (HBSC) cross-sectional survey. The HBSC study is an international network supported by the World Health Organization that collects data in more than 40 countries in Europe and North America. The survey is conducted every 4 years with the aim of increasing knowledge about health-related behaviors, lifestyles and developmental contexts of young people.

Participants of the present study come from a representative sample of school-aged children aged 13–16 years residing in Spain, who were selected for the 2014 edition of the HBSC study using a random multi-stage sampling stratified by conglomerates, representative by age, area of residence (rural or urban), type of school (public or private) and region (Spain has 19 regions) (Moreno et al., 2016). Participants were recruited from a database of schools published by the Spanish Ministry of Education. Those centers that refused to participate in the study were substituted for other centers, also selected randomly within the same stratum. The final student participation rate was 87%.

For the purpose of this article, terciles were used to identify adolescents scoring high (upper tercile) and low (lower tercile) in the scales for Global Health Score (GHS) and the Quality of the Parent-Child Relationship (QPCR) (described later in the section on instruments).

Despite the limitations of categorizing quantitative variables (Preacher et al., 2005), dividing them into three groups in order to identify their extremes is supported by three reasons: firstly, by the essence of the construct itself, since “resilience is never directly measured, but instead is indirectly inferred based on evidence of the two subsumed constructs” (“adversity” and “positive adaptation”; Luthar et al., 2015, p. 248); secondly, it is consistent with literature that identifies both resilient and vulnerable subjects as extreme groups in unfavorable and favorable circumstances, respectively, but whose results in adjustment indicators are not consistent with their circumstances (Luthar et al., 2000; Masten, 2014); and lastly, from a purely methodological perspective, because, as DeCoster et al. (2009) argues, categorization is advised when focusing on the extreme groups since it allows for identification of groups of subjects based on conceptual definitions.

Based on the four combinations resulting from this division 1753 adolescents were selected from the total of 3845 studied (see Table 2). In the selected sample, 45.8% are boys and 54.2% are girls, with a mean age of 14.62 years (*SD* = 1.11). Additionally, 62.7% attended public schools and 37.3% private, with 54.1% living in urban areas and 54.9% in rural areas.

**Table 2:** **Sample subgroups according to their tercile position in the global health and the quality of parent–child relationship scores (the four groups examined in the present study are highlighted in bold)**.

		**Global Health Score (GHS)**		
		**Tercile 1 (low)**	**Tercile 2 (medium)**	**Tercile 3 (upper)**
**Quality of Parent-Child Relationships (QPCR)**	**Tercile 1 (low)**	**726**	386	**172**
	**Tercile 2 (medium)**	402	505	398
	**Tercile 3 (upper)**	**150**	401	**705**

Therefore, following the classification criteria for adaptation status developed by classic research (Tiêt and Huizinga, 2002), the sample was classified in four groups, defined as follows: resilient adolescents (tercil 1 in QPCR and tercil 3 in GHS), maladaptative adolescents (tercil 1 in CRPF and tercil 1 in GHS), vulnerable adolescents (tercil 3 in CRPF and tercil 1 in GHS) and competent adolescents (tercil 3 in QPCR and tercil 3 in GS).

### Instruments

The variables were assessed using the 2014 Spanish HBSC Questionnaire, which included questions about lifestyles, positive health and characteristics of the principal developmental contexts (family, peers, and school) in adolescence. The instrument is comprised of an extensive series of mandatory questions, optional packages and questions that cover specific national interests (Roberts et al., 2009). The complete questionnaire is revised and improved for each edition of the study (for the last edition, see Inchley et al., 2016). For the present paper, key measures of quality of parent-child relationship and health, as well as sociodemographic, school and peer contexts, lifestyle, and psychological and socioeconomic variables were selected from the Spanish version of the 2014 HBSC survey.

Firstly, the following two measures were used to derive the classification in groups (maladaptative, resilient, vulnerable, and competent) that acts as the dependent variable.

1. Global Health Score (GHS). This measure is based on 20 items related to the variables: life satisfaction, self-rated health, health-related quality of life and psychosomatic complaints. The details of these instruments can be consulted in Table 3. The GHS is a score with a mean of 50 and standard deviation of 10 that has shown good fit indices (NNFI = 0.98, CFI = 0.99, RMSEA = 0.03), as well as good reliability and validity (Ramos et al., 2010). This measure assesses the adolescent's physical, psychological and social wellbeing, following the most widely used and currently accepted definition of health, i.e., the definition proposed by the World Health Organization (WHO, 1948). As previously described, terciles were used in the present study to classify the adolescents in three groups according to this measure of global health.
2. Factorial score on the Quality of Parent-Child Relationship (QPCR), with a mean of 5 and a standard deviation of 2. This score is an adaptation of the measure developed by García-Moya et al. (2013a), that consists of the following three indicators: perceived affection, ease of communication with parents and satisfaction with family relations. The details of these instruments can be consulted in Table 3. The factorial score on the QPCR showed good fit indices (NNFI = 0.99, CFI = 0.99, RMSEA = 0.02) has been considered a useful tool in global assessments of the relationships between parents and children according to the adolescents' perception (García-Moya et al., 2013a). As previously mentioned, terciles were used in the present study to classify adolescents in three groups according to the quality of their parent-child relationship.

**Table 3:** **Dependent variables and instruments used for their assessment in the present study**.

Global Health Score (GHS)	Life satisfaction	It was measured by the Cantril's Ladder (Cantril, 1965), with the question: “Here is a picture of a ladder. The top of the ladder ‘10’ is the best possible life for you and the bottom ‘0’ is the worst possible life for you. In general, where on the ladder do you feel you stand at the moment? Tick the box next to the number that best describes where you stand.” This variable represents the global perception adolescents have of their lives, *from 0 to 10*. Level of measurement: quantitative variable.
	Self-reported health	A single item asked the adolescent to consider their health at that moment, with their response fitting to one of the following four options: *excellent, good, passable*, or *poor* (Idler and Benyamini, 1997). This measure has been validated for quantitative use (Silventoinen et al., 2007). Level of measurement: ordinal variable.
	Health-related quality of life	It was measured with the Kidscreen instrument designed for a population between the ages of 8 to 18. Specifically the Kidscreen-10 version was used, which provides a global, health-related quality of life index with 10 items covering physical, psychological and social aspects (Ravens-Sieberer et al., 2001). These items, which show a Cronbach's alpha of 0.83, refer to feeling well and fit, full of energy, sad, lonely, having enough time for themselves, doing things they want in their free time, receiving fair treatment from their parents, having a good time with friends, getting on well at school and being able to pay attention/concentrate. Items were answered on a 5-point Likert scale, from 1, *never*, to 5*, always*. Level of measurement: continuous variable.
	Psycho-somatic complaint	It was measured with the HBSC-symptom checklist. It measures two aspects (Ravens-Sieberer et al., 2008): psychological complaint (nervousness, feeling low, irritability and sleeping problems) and somatic manifestations (headache, stomach-ache, back ache, and feeling dizzy), with a Cronbach's alpha of 0.83. These 8 items were answered on a 5-point Likert scale: *about every day, more than once a week, about every week, about every month*, and *rarely or never*. Level of measurement: continuous variable.
Factorial Score on the Quality of Parent-Child Relationship (QPCR)	Perceived affection	This variable was assessed by means of the 4-item subscale of the Parental Bonding Inventory-Brief Current form (PBI-BC; Klimidis et al., 1992), with the aim of determining if the parents showed to be warm and supportive toward their children. This dimension includes the following items repeated for the mother and the father: “helps me as much as I need,” “is loving,” “understand my problems and worries,” and “makes me feel better when I'm upset.” An average score from 0, *never*, to 2, *almost always*, was obtained from this scale, with a Cronbach's alpha of 0.85. Level of measurement: continuous variable.
	Ease of communication with parents	Participants were asked: “how easy is it for you to talk to your father about things that really bother you?” and “how easy is it for you to talk to your mother about things that really bother you?” (these questions were created by the HBSC study). An average score on ease of communication with parents was obtained that ranged from 1, *very difficult*, to 4, *very easy*. Level of measurement: ordinal variable.
	Satisfaction with family relations	This variable was measured by means of an item based on Cantril's Ladder (1965): “in general, how satisfied are you with the relationships in your family?” A quantitative score was obtained that ranged *from 0 “we have very bad relationships in our family” to 10 “We have very good relationships in our family*.” Level of measurement: quantitative variable.

In addition, the independent variables were selected in line with the aims of this study and assessed by means of several instruments that were part of the 2014 HBSC Questionnaire, explained above. The details of these instruments are presented in Table 4.

**Table 4:** **Independent variables and instruments used for their assessment in the present study**.

Sociodemographic variables	Sex		Boy and girl. Level of measurement: categorical variable.
	Age		13–16 years. Level of measurement: continuous variable.
	Type of educational center		Public and private. Level of measurement: categorical variable.
	Habitat		Urban and rural. Level of measurement: categorical variable.
School context variables	Perceived academic achievement		They were asked: “in your opinion, what does your teacher think about your school performance compared to your classmates” (this question was created by the HBSC study). This question is answered on a 4-point Likert scale, ranging from 1, *below average*, to 4, *very good*. Level of measurement: quantitative variable.
	Feelings toward school		The following question: “how do you feel about school at the present?” (this question was created by the HBSC study). Four response options were available on a 4-point Likert scale from 1, *I don't like it at all*, to 4, *I like it a lot*. Level of measurement: continuous variable.
	Teacher support		It was assessed by means of the following three items: “I feel that my teachers accept me as I am,” “I feel that my teachers care about me as a person,” and “I feel a lot of trust in my teacher,” with a Cronbach's alpha of 0.84. Items were answered on a 5-point Likert scale, from 1, *I completely disagree*, to 5*, I completely agree* (Torsheim et al., 2000). Level of measurement: continuous variable.
Peer context variables	Perceived social support		It was assessed by means of the Multidimensional Scale of Perceived Social Support (MSPSS; Zimet et al., 1988). This scale consists of the following four items: “my friends really try to help me,” “I can count on my friends when things go wrong,” “I have friends with whom I can share my joys and sorrows,” and “I can talk about my problems with my friends,” Items are answered on a 7-point Likert scale, from *completely disagree* (1) to *completely agree* (7), with a Cronbach's alpha of 0.98. Level of measurement: continuous variable.
	Models of behavior in the peer group		It was assessed by means on a scale developed by the HBSC study network and validated by Gaspar de Matos et al. (unpublished manuscript). Adolescents were asked about the frequency of 8 different behaviors in their group of friends, including both positive (such as “do well in school,” “participate in sports activities with other kids,” “participate in cultural activities other than sports” and “get along well with parents”) and negative (such as “smoke cigarettes,” “drink alcohol,” “get drunk,” and “consume drugs to get high”) behaviors. Items were answered on a Likert scale from 1, *never or almost never*, to 3, *often*, with a Cronbach's alpha of 0.70. The items corresponding to negative behaviors were reverse-coded, so that a higher score on this scale represents a higher presence of positive models of behavior in the peer group. Level of measurement: continuous variable.
	Satisfaction with friendships		Measure adapted by the HBSC network from the Cantril's Ladder on life satisfaction scaled *from 0 to 10* (Cantril, 1965), but referring specifically to satisfaction with friendships. Level of measurement: quantitative variable.
	Having been bullied		It was assessed by means of the Revised Bully/Victim Questionnaire (Olweus, 1996). The response options ranged from 1, *I haven't been bullied in school in the past 2 months*, to 5*, multiple times a week*. Level of measurement: quantitative variable.
	Having bullied others		Also assessed by means of the Olweus (1996) questionnaire and with similar response options. Level of measurement: quantitative variable.
Lifestyle variables	Eating habits	Breakfast frequency	Adolescents were asked how many days a week they typically ate breakfast (something more than a glass of milk or juice), with the corresponding response values ranging from 1 to 7 days. In addition, they also answered questions on how many times a week they typically ate two specific types of foods: fruits and snacks. These questions were created by the HBSC study. The response options varied from 1*, never*, to 7, *every day, more than once*. Level of measurement: quantitative variable.
		Fruit consumption		
		Snack consumption		
	Physical activity	MVPA	Adolescents were asked about their level of Moderate to Vigorous Physical Activity (MVPA), as indicated by the number of days in which they felt physically active during a total of at least 60 min a day over the last 7 days. The response options ranged from 0 to 7 days (Prochaska et al., 2001). In addition, they were asked about their level of Vigorous Physical Activity (VFA), assessed in the HBSC study by the frequency with which the adolescents, in their free time outside of school hours, engaged in some type of physical activity that made them sweat or out of breath. The response options on a Likert scale ranged from 1, *never*, to 7*, every day*. Level of measurement: quantitative variable.
		VFA		
	Dental hygiene		Adolescents were asked how often they brushed their teeth (these questions were created by the HBSC study), with the following response options: *never; less than once a week; at least once a week but not daily; once a day;* and *more than once a day*. Level of measurement: quantitative variable.
	Substance use	Tobacco use	Three questions referring to the frequency of substance use over the past 30 days were included. These items have been adapted from the questions included in the European School Survey Project on Alcohol and Other Drugs (Hibell et al., 2000). Specifically, adolescents were asked about the number of days, out of past 30 days, in which they had smoked cigarettes, had drank alcohol and had smoked cannabis (hash or marijuana, “joints”). These items were answered on a 7-point Likert scale, from 1, *never*, to 7*, 30 days*. Level of measurement: quantitative variable.
		Alcohol use		
		Cannabis use		
Psychological variables	Sense of coherence		This construct was assessed by means of the SOC-13 scale (Antonovsky, 1987). It consists of 13 items, such as “has it happened in the past that you were surprised by the behavior of people whom you thought you knew well?,” and “how often do you have the feeling that there's little meaning in the things you do in your daily life?.” Questions are answered on a 7-point Likert scale, with a Cronbach's alpha of 0.77. The SOC-13 scale has shown good reliability and validity in different countries (Lindström and Eriksson, 2010). Level of measurement: continuous variable.
	Emotional regulation		It was assessed by means of the impulsiveness/emotion-control subscale from the reduced version of the Emotion Regulation Index for Children and Adolescents scale (ERICA; MacDermott et al., 2010). This subscale comprises 8 items (for example, “I have angry outbursts,” “I have trouble waiting for something I want”) and it is answered on a 5-point Likert scale, from 1, *totally agree*, to 5, *totally disagree*. The Cronbach's alpha was of 0.84. Level of measurement: continuous variable.
	Curiosity and Exploration		It was assessed by means of the Curiosity and Exploration Inventory-II (Kashdan et al., 2009). It is a scale with 10 items (some examples are: “I am at my best when doing something that is complex or challenging,” “I am the kind of person who embraces unfamiliar people, events, and places,” or “I like to do things that are a little frightening”) with 5 response options on a Likert scale, from 1, *a little or none*, to 5, *a lot*. The Cronbach's alpha was of 0.87. Level of measurement: continuous variable.
	Perceived body image		It was assessed with an item created for the HBSC study. Specifically, they are asked “do you think your body is…?” and the response options on a 5-point Likert scale ranged from 1, *much too fat*, to 5*, much too thin*. Level of measurement: ordinal variable.
	Satisfaction with body image		It was assessed by means of the subscale of feelings and attitudes toward the body of the Body Investment Scale (BIS; Orbach and Mikulincer, 1998). This subscale consists of 6 items (“I am frustrated with my physical appearance,” “I am satisfied with my appearance,” “I hate my body,” “I feel comfortable with my body,” “I feel anger toward my body,” and “I like my appearance in spite of its imperfections”), and is answered on a 5-point Likert scale, from 1, *totally agree*, to 5*, totally disagree*. The Cronbach's alpha was of 0.89. Level of measurement: continuous variable.
Socioeconomic variables	Father educational level		Father's and mother's educational level and perceived family wealth were assessed with three questions created by the HBSC study. Educational level was measured on a 4-point Likert scale, from 1, *never studied (does not know how to read nor write, or does so with difficulty*) to 4, *university studies, either finished or unfinished*. The level of perceived family wealth was assessed by asking “how well off do you think your family is?.” The question was answered on a 5-point Likert scale, from 1, *not at all well off*, to 5, *very well off*. Level of measurement: quantitative variable.
	Mother educational level			
	Perceived family wealth			

### Procedure

New information and communication technologies (ICT), based on a CAWI (Computer-Assisted Web Interviewing) model, were used in the data collection process. The data was always collected in the school setting, under the supervision of teachers. In those schools with internet-connection problems or problems with the condition or number of computers, members of the research team traveled personally to those schools to collect data using tablets. Ultimately, the guided computerized procedure has the advantage of immediately receiving and incorporating the students' responses in the database, thus reducing the possible errors from the data entry process, as well as helping to maintain the anonymity of the responses.

In all of the schools, after contacting via telephone with the head teacher, deputy head teacher or school counselor, instructions were given to the teachers who would be supervising the classroom when the adolescents responded to the questionnaires. On the other hand, instructions for the students were included at the beginning of the questionnaire to guarantee homogeneity amongst all the participants.

Ultimately, data collection complied with the three requirements dictated by the HBSC international protocol (Roberts et al., 2009): students themselves answered the questionnaires; anonymity was guaranteed; and the questionnaires were completed at school under the supervision of instructed staff.

### Statistical analysis

Firstly, bivariate analyses including chi-square and ANOVA (with Bonferroni test for multiple comparisons) were used to compare the four groups of adolescents (maladaptative, resilient, competent, and vulnerable) in each one of the independent variables (sociodemographic, school context, peer context, lifestyle, psychological, and socioeconomic variables). This analysis corresponds to the research question 1. Also, Crammer's *V* and Cohen's *d* were calculated to determine the effect size, with the following cut-off points: 0–0.19 = negligible, 0.20–0.49 = small, 0.50–0.79 = medium, 0.80 and above = high (Cohen, 1988).

Secondly, separate binary logistic regression analyses were carried out for resilience and vulnerability, with adaptation status (resilient vs. maladaptative -research question 2- and vulnerable vs. competent -research question 3-, respectively) as the dependent variables, and the different sets of variables analyzed (demographic, school context, peer context, lifestyle, psychological, and socioeconomic variables) as predictor variables. The predictive capacity of each set of variables (controlling for significant demographic variables) was calculated using the Nagelkerke R^2^. Afterwards, a final model including only significant variables in previous analysis was estimated. The odds ratio (OR) and its confidence interval at the 95% level (95% CI) was calculated for each examined predictor, establishing the statistical significance as *p* < 0.05 for each variable.

Statistical analyses were conducted using the IBM SPSS Statistics 22.0 software.

## Results

### Research question 1. comparisons among the four adaptation groups: maladaptative, resilient, competent, and vulnerable adolescents

This first subsection focuses on the comparisons among maladaptative, resilient, competent and vulnerable adolescents in all variables of this study. The comparisons of these groups show significant differences (*p* < 0.001, *V* = 0.231, medium effect size) in the distribution of gender. Table 5 shows that the maladaptative and vulnerable groups have a greater proportion of girls. However, comparisons between the four groups are not significant neither for educational center (*p* = 0.067, *V* = 0.087, negligible effect size) nor habitat (*p* = 0.145, *V* = 0.051, negligible effect size).

**Table 5:** **Percentage of maladaptative, resilient, competent and vulnerable adolescents in relation to the sex (boys and girls), the type of educational center (public and private) and the habitat (urban and rural)**.

		**Maladaptative (%)**	**Resilient (%)**	**Competent (%)**	**Vulnerable (%)**
**Sex**	Boys	33.81	60.93	57.47	40.00
	Girls	66.19	39.07	42.53	60.00
**Type of educational center**	Public	66.59	61.26	62.34	67.14
	Private	33.41	38.74	37.66	32.86
**Habitat**	Urban	55.16	52.32	50.81	55.36
	Rural	44.84	47.68	49.19	44.64

Table 6 shows the distribution of the maladaptative, resilient, competent and vulnerable groups in the age, school context, peer context, lifestyle, psychological, and socioeconomic variables. The mean comparisons of the contrasts between pairs of groups can be consulted in Table 7.

**Table 6:** **Descriptive statistics of the age, school context, peer context, lifestyle, psychological and socioeconomic variables between maladaptative, resilient, competent and vulnerable adolescents**.

**Variables**		**Maladaptative**		**Resilient**		**Competent**		**Vulnerable**	
		***M***	***SD***	***M***	***SD***	***M***	***SD***	***M***	***SD***
Age		14.92	1.07	14.52	1.10	14.28	1.10	14.84	1.10
**School context**	Perceived academic achievement	2.40	0.80	2.87	0.81	3.08	0.74	2.53	0.77
	Feelings toward school	2.37	0.88	2.67	0.86	3.01	0.88	2.60	0.87
	Perceived teacher support	3.19	0.88	3.73	0.90	4.09	0.84	3.60	0.88
**Peer context**	Perceived social support	5.18	1.63	5.81	1.44	6.11	1.41	5.70	1.46
	Models of behavior	2.91	0.43	3.08	0.42	3.16	0.42	2.99	0.42
	Satisfaction with friendships	7.78	1.85	8.87	1.28	9.05	1.35	8.27	1.77
	Having been bullied	1.32	0.78	1.09	0.42	1.14	0.53	1.23	0.73
	Having bullied others	1.32	0.74	1.24	0.58	1.14	0.51	1.19	0.59
**Lifestyles**	Breakfast frequency	5.05	2.36	6.16	1.68	6.44	1.47	5.77	2.02
	Fruit consumption	4.22	1.72	4.59	1.65	4.94	1.63	4.39	1.58
	Snack consumption	3.59	1.20	3.63	1.15	3.47	1.17	3.62	1.11
	MVPA	4.63	1.91	6.11	1.91	6.12	1.75	4.69	1.82
	VFA	4.51	1.67	5.38	1.52	5.42	1.37	4.50	1.67
	Dental hygiene	4.46	0.83	4.52	0.78	4.69	0.55	4.61	0.64
	Tobacco use	1.59	1.51	1.22	0.98	1.08	0.58	1.26	1.09
	Alcohol use	1.67	1.14	1.47	1.06	1.20	0.63	1.48	1.02
	Cannabis use	1.22	0.84	1.09	0.53	1.04	0.37	1.07	0.48
**Psychological variables**	Sense of coherence	3.72	0.78	4.72	0.81	5.41	0.87	4.32	0.75
	Emotional regulation	2.79	0.73	3.19	0.81	3.63	0.88	3.14	0.86
	Curiosity and exploration	2.85	0.81	3.39	0.84	3.48	0.91	3.03	0.84
	Perceived body image	2.55	0.91	2.98	0.67	3.01	0.62	2.71	0.82
	Satisfaction with body image	3.20	1.01	4.23	0.88	4.46	0.73	3.57	0.85
**Socioeconomic variables**	Father educational level	2.79	0.75	2.90	0.81	3.03	0.78	2.97	0.79
	Mother educational level	2.94	0.79	3.03	0.80	3.20	0.79	2.97	0.84
	Perceived family wealth	2.94	0.50	3.11	0.48	3.09	0.43	3.00	0.53

*MVPA, Moderate to Vigorous Physical Activity; VPA, Vigorous Physical Activity*.

**Table 7:** **Mean comparisons test (ANOVA with Bonferroni correction for multiple comparisons and effect size) of age, school context, peer context, lifestyle, psychological and socioeconomic variables between maladaptative, resilient, competent, and vulnerable adolescents**.

**Variables**		**Maladaptative/Resilient**	**Maladaptative/Competent**	**Maladaptative/Vulnerable**	**Resilient/Competent**	**Resilient/Vulnerable**	**Competent/Vulnerable**
Age		<0.001^*^	<0.001^**^	>0.999	0.003^*^	0.003^*^	<0.001^**^
**School context**	Perceived academic achievement	<0.001^**^	<0.001^***^	0.483	0.012^*^	<0.001^*^	<0.001^**^
	Feelings toward school	<0.001^*^	<0.001^**^	0.020^*^	<0.001^*^	>0.999	<0.001^*^
	Perceived teacher support	<0.001^**^	<0.001^***^	<0.001^**^	<0.001^*^	0.948	<0.001^**^
**Peer context**	Perceived social support	<0.001^*^	<0.001^**^	0.001^*^	0.118	>0.999	0.015^*^
	Models of behavior	<0.001^*^	<0.001^**^	0.304	0.123	0.324	<0.001^*^
	Satisfaction with friendships	<0.001^**^	<0.001^**^	0.003^*^	>**0.999**	0.005^*^	<0.001^**^
	Having been bullied	<0.001^*^	<0.001^*^	0.969	>0.999	0.329	0.685
	Having bullied others	>0.999	<0.001^*^	0.181	0.312	>0.999	>0.999
**Lifestyles**	Breakfast frequency	<0.001^**^	<0.001^**^	<0.001^*^	0.538	0.468	0.001^*^
	Fruit consumption	0.048	<0.001^*^	>0.999	0.083	>0.999	0.002^*^
	Snack consumption	>0.999	0.290	>0.999	0.571	>0.999	0.884
	MVPA	<0.001^**^	<0.001^***^	>0.999	>0.999	<0.001^**^	<0.001^***^
	VFA	<0.001^**^	<0.001^**^	>0.999	>0.999	<0.001^**^	<0.001^**^
	Dental hygiene	>0.999	<0.001^*^	0.115	0.025^*^	>0.999	>0.999
	Tobacco use	0.001^*^	<0.001^*^	0.008^*^	0.867	>0.999	0.466
	Alcohol use	0.071	<0.001^**^	0.138	0.005^*^	>0.999	0.007^*^
	Cannabis use	0.102	>0.999	0.053	>0.999	>0.999	>0.999
**Psychological variables**	Sense of coherence	<0.001^***^	<0.001^***^	<0.001^**^	<0.001^***^	<0.001^**^	<0.001^***^
	Emotional regulation	<0.001^**^	<0.001^***^	<0.001^*^	<0.001^**^	>0.999	<0.001^**^
	Curiosity and exploration	<0.001^**^	<0.001^**^	0.257	>0.999	0.016^*^	<0.001^**^
	Perceived body image	<0.001^**^	<0.001^**^	0.139	>0.999	0.011^*^	<0.001^*^
	Satisfaction with body image	<0.001^***^	<0.001^***^	<0.001^*^	0.017^*^	<0.001^**^	<0.001^***^
**Socioeconomic variables**	Father educational level	0.502	<0.001^*^	0.045^*^	0.301	>0.999	>0.999
	Mother educational level	0.827	<0.001^*^	>0.999	0.089	>0.999	0.009^*^
	Perceived family wealth	<0.001^*^	<0.001^*^	0.818	>0.999	0.223	0.184

MVPA, Moderate to Vigorous Physical Activity; VPA, Vigorous Physical Activity. Effect size interpretation: 0–0.19 = negligible (–), 0.20–0.49 = small (

* ), 0.50–0.79 = medium (

** ), 0.80 and above = high (

*** *). The bold values indicates (small, medium, or high) effect size values*.

Regarding age, older adolescents fell into the maladaptive and vulnerable categories, followed by the resilient adolescents and finally, the youngest fell into the category of competent adolescents.

With respect to school, the competent adolescents show higher perception of academic achievement than the resilient adolescents, who in turn have a higher perception than the maladaptative and vulnerable adolescents. In relation to feeling toward school, the competent adolescents have the most positive feelings toward school and the highest perception of teacher support, followed by the resilient and vulnerable adolescents and, finally, the maladaptative adolescents.

In their peer relationships, the competent and resilient adolescents show the highest perception of social support, followed by the vulnerable adolescents and, finally, the maladaptative adolescents. The resilient and competent adolescents have a higher rate of positive models of behavior in their peer group than the maladaptative adolescents, with the vulnerable adolescents falling in the middle. Likewise, resilient and competent adolescents have higher satisfaction with their friendships than the vulnerable adolescents, and this group shows more satisfaction than maladaptative adolescents. In relation to bullying, the maladaptative adolescents show a higher likelihood to have been bullied and to have bullied others than the other groups (resilient, competent, and vulnerable adolescents).

Regarding lifestyles, the competent and resilient adolescents eat breakfast more days a week, followed by the vulnerable adolescents and, finally, the maladaptative adolescents. The resilient and competent adolescents eat fruit more frequently than the maladaptative adolescents do (the vulnerable adolescents show an intermediate score between the maladaptive and resilient adolescents). Likewise, resilient and competent adolescents do more physical activity (moderate to vigorous and vigorous) than the maladaptative and vulnerable adolescents. The competent adolescents brush their teeth more frequently than the maladaptative and resilient adolescents (the vulnerable adolescents show an intermediate score between the competent and resilient adolescents). In relation to tobacco, the maladaptative adolescents show higher use than the other three groups. However, the competent adolescents show lower alcohol use than all the others.

The analyses of psychological variables show differences in sense of coherence among the four groups of adolescents. Ordered from the highest to lowest score they are: competent, resilient, vulnerable, and maladaptative adolescents. In relation to emotional regulation, the competent adolescents have the highest score, followed by the resilient and vulnerable adolescents and, finally, the maladaptative adolescents. The resilient and competent adolescents present more curiosity and exploration and they see themselves as less obese than the maladaptative and vulnerable adolescents. In addition, there are differences among the four groups regarding satisfaction with body image. Ordered from highest to lowest they are: competent, resilient, vulnerable and maladaptative adolescents. Lastly, significant differences are found in parents' education, showing that the educational level of the competent and vulnerable adolescents' fathers is higher than that of the fathers of maladaptative adolescents. However, the educational level of the competent adolescents' mothers is higher than that of the mothers of maladaptative and vulnerable adolescents. The resilient adolescents show an intermediate score between maladaptive and vulnerable adolescents for both the father and mother's education. There are significant differences in perceived family wealth, being higher in the resilient and competent adolescents than it is in the maladaptative ones (in this case the vulnerable adolescents are situated between the competent and maladaptive adolescents).

### Research question 2. the study of the resilient adolescents

This second subsection focuses on those adolescents who, despite having low-quality parent-child relationships have a high global health score, that is to say, the resilient group (4.5% of the global sample and 13.4% of the participants classified as low-quality in parent-child relationship). This group of adolescents are compared with those which, having a low-quality parent-child relationship, have a low global health score, that is to say, the maladaptative group (18.9% of the global sample and 56.5% of the sample with low-quality parent-child relationships).

The results of the logistic regression analyses using the group of resilient adolescents as the reference value are shown below. Specifically, six models have been estimated, one for each group of independent variables (although sex and age have been included in all of them to prevent them to become confounding variables). Additionally, a global model is shown at the end, including only those variables that were found to be significant in previous models.

As can be seen in the first row of Table 8, although model 1 explained 10.8% of the total variability, being significant the variables sex and age (specifically, boys and younger adolescents have a higher probability of being resilient), using these demographic variables only the percentage of well-classified adolescents was 0%.

**Table 8:** **Logistic regression models on resilience by demographic, school context, peer context, lifestyle, psychological and socioeconomic variables**.

		**Model 1-OR (95%-CI) Demographic variables**	**Model 2-OR (95%-CI) School context**	**Model 3-OR (95%-CI) Peer context**	**Model 4-OR (95%-CI) Lifestyles**	**Model 5-OR (95%-CI) Psychological variables**	**Model 6-OR (95%-CI) Socioeconomic variables**	**Model 7-OR (95%-CI) Global**
	**Variables**	***R*^2^ = 0.108 (80.5/0.0%)**	***R*^2^ = 0.228 (83.2/22.1%)**	***R*^2^ = 0.238 (82.4/19.2%)**	***R*^2^ = 0.247 (83.0/25.6%)**	***R*^2^ = 0.370 (87.2/30.4%)**	***R*^2^ = 0.131 (80.2/3.5%)**	***R*^2^ = 0.518 (89.3/51.5%)**
**Demographic variables**	Sex (ref. girls)	3.23^**^ (2.27–4.58)	3.57^**^ (2.45–5.21)	3.60^**^ (2.46–5.27)	2.21^**^ (1.47–3.32)	1.35 (0.79–2.33)	3.23 (2.27–4.61)	1.35 (0.73–2.48)
	Age	0.70^**^ (0.59–0.83)	0.73^**^ (0.62–0.87)	0.72^**^ (0.60–0.87)	0.74^**^ (0.62–0.90)	0.72 (0.50–1.04)	0.69 (0.58–0.81)	0.88 (0.59–1.32)
	Type of educational center (ref. public)	1.40 (0.98–1.99)	NA	NA	NA	NA	NA	NA
	Habitat (ref. urban)	1.04 (0.74–1.47)	NA	NA	NA	NA	NA	NA
**School context**	Perceived academic achievement	NA	1.83^**^ (1.44–2.33)	NA	NA	NA	NA	1.64^**^ (1.13–2.38)
	Feelings toward school	NA	1.19 (0.96–1.50)	NA	NA	NA	NA	NA
	Perceived teacher support	NA	1.19^**^ (1.11–1.29)	NA	NA	NA	NA	1.19^**^ (1.06–1.33)
**Peer context**	Perceived social support	NA	NA	1.04 (1.00–1.08)	NA	NA	NA	NA
	Models of behavior	NA	NA	1.07^*^ (1.01–1.14)	NA	NA	NA	1.04 (0.95–1.15)
	Satisfaction with friendships	NA	NA	1.50^**^ (1.26–1.79)	NA	NA	NA	1.31^*^ (1.02–1.68)
	Having been bullied	NA	NA	0.53^**^ (0.33–0.84)	NA	NA	NA	0.82 (0.42–1.62)
	Having bullied others	NA	NA	0.92 (0.68–1.25)	NA	NA	NA	NA
**Lifestyles**	Breakfast frequency	NA	NA	NA	1.24^**^ (1.12–1.38)	NA	NA	1.33^**^ (1.13–1.56)
	Fruit consumption	NA	NA	NA	1.02 (0.91–1.15)	NA	NA	NA
	Snack consumption	NA	NA	NA	1.07 (0.90–1.26)	NA	NA	NA
	MVPA	NA	NA	NA	1.37^**^ (1.22–1.54)	NA	NA	1.24^**^ (1.05–1.45)
	VPA	NA	NA	NA	1.13 (0.97–1.30)	NA	NA	NA
	Dental hygiene	NA	NA	NA	1.17 (0.91–1.50)	NA	NA	NA
	Tobacco use	NA	NA	NA	0.88 (0.68–1.13)	NA	NA	NA
	Alcohol use	NA	NA	NA	0.92 (0.75–1.13)	NA	NA	NA
	Cannabis use	NA	NA	NA	0.89 (0.61–1.31)	NA	NA	NA
**Psychological variables**	Sense of coherence	NA	NA	NA	NA	3.18^**^ (2.14–4.73)	NA	2.74^**^ (1.84–4.10)
	Emotional regulation	NA	NA	NA	NA	1.01 (0.96–1.06)	NA	NA
	Curiosity and exploration	NA	NA	NA	NA	1.07^**^ (1.03–1.11)	NA	1.05^**^ (1.01–1.10)
	Perceived body image	NA	NA	NA	NA	1.17 (0.82–1.66)	NA	NA
	Satisfaction with body image	NA	NA	NA	NA	1.83^**^ (1.31–2.56)	NA	1.84^**^ (1.31–2.58)
**Socio econom**.	Father educational level	NA	NA	NA	NA	NA	1.08 (0.83–1.39)	NA
	Mother educational level	NA	NA	NA	NA	NA	1.06 (0.82–1.36)	NA
	Perceived family wealth	NA	NA	NA	NA	NA	1.98^**^ (1.37–2.85)	2.83^**^ (1.47–5.44)

MVPA, Moderate to Vigorous Physical Activity; VPA, Vigorous Physical Activity; OR, Odds Ratio (95% CI = Confidence Interval at the 95% level); R^2^ = Model explained variance (% correctly-classified total/% correctly-classified resilient group); NA, not applicable.

* p < 0.05,

** *p < 0.01*.

In model 2, concerning school context, the explained variance is 22.8 and 22.1% of the resilient adolescents are correctly classified. In this case, those adolescents with a higher perception of academic achievement, with an OR of 1.83 (95% *CI* = 1.44–2.33), and those with higher teacher support (*OR* = 1.19, 95% *CI* = 1.11–1.29), have a higher likelihood of being resilient.

In model 3, which includes the variables of peer context, the predictive capacity is 23.8%, with 19.2% of the adolescents in the resilient group being correctly classified. Significant variables in this model are models of behavior, satisfaction with friendships and being a victim of bullying. Adolescents who are more satisfied with their friendships are 1.5 times more likely to be resilient (95% *CI* = 1.26–1.79), whereas those that were victims of bullying more often have a lower likelihood of being resilient (*OR* = 0.53, 95% *CI* = 0.33-0.84). Likewise, those adolescents with a group of friends providing better models of behavior also show a higher likelihood of being resilient (*OR* = 1.07, 95% *CI* = 1.01–1.14).

Model 4 is devoted to variables related to lifestyles and shows a level of explained variance of 24.7%, with 25.6% of the resilient adolescents being correctly classified. Only two variables in this model are significant: breakfast frequency and moderate to vigorous physical activity. Specifically, those adolescents that engage in higher levels of moderate to vigorous physical activity increase their likelihood of being resilient in 1.37 times (95% *CI* = 1.22–1.54). Additionally, those adolescents who have breakfast more regularly are more likely to be resilient (*OR* = 1.24, 95% *CI* = 1.12–1.38).

Model 5 includes the psychological variables. Among the six specific models, this model shows the highest level of explained variance, which reaches 37% (30.4% of the adolescents in resilient group are correctly classified). The significant variables in this model are: sense of coherence, curiosity and exploration and satisfaction with body image. Sense of coherence stands out for its high OR, which is 3.18 (95% *CI* = 2.14–4.73), meaning that those adolescents with higher scores in this psychological construct have the highest likelihood of being resilient. Next, adolescents with a higher satisfaction with their body image are 1.83 times more likely to be resilient (*OR* = 1.83, 95% *CI* = 1.31–2.56). Lastly, those adolescents with higher scores in curiosity and exploration have a higher likelihood of being resilient (*OR* = 1.07, *CI* 95% = 1.03–1.11).

Model 6, referring to the socioeconomic variables, shows a lower predictive capacity than the previous models (13.1%), with only 3.5% of resilient adolescents being correctly classified. The only significant variable in this model is perceived family wealth, meaning that those who perceive a higher family wealth are 1.98 times more likely to be resilient (95% *CI* = 1.37–2.85).

Finally, in model 7 or the global model (the one which includes only the significant variables from previous models), the results show that the variables sex, age, models of behavior in the peer group and being a victim of bullying loose predictive capacity. Therefore, the global model includes the following nine variables: perceived academic achievement, perceived teacher support, satisfaction with friendships, breakfast frequency, moderate to vigorous physical activity, sense of coherence, curiosity and exploration, satisfaction with body image and perceived family wealth. This model stands out for its high predictive capacity, surpassing 50% of the explained variance (specifically, 51.8%). Additionally, there are a notably high proportion of correctly-classified resilient adolescents, specifically, 51.5%.

The most influential independent variables, with ORs higher than 2, are perceived family wealth (*OR* = 2.83, 95% *CI* = 1.47–5.44) and sense of coherence (*OR* = 2.74, 95% *CI* = 1.84–4.10). Satisfaction with body image (*OR* = 1.84, 95% *CI* = 1.31–2.58) and perceived academic achievement (*OR* = 1.64, 95% *CI* = 1.13–2.38) also stand out. Lastly, more modest contributions were found for breakfast frequency (*OR* = 1.33, 95% *CI* = 1.13–1.56), satisfaction with friendships (*OR* = 1.31, 95% *CI* = 1.02–1.68), frequency of moderate to vigorous physical activity (*OR* = 1.24, 95% *CI* = 1.05–1.45), teacher support (*OR* = 1.19, 95% *CI* = 1.06–1.33) and curiosity and exploration (*OR* = 1.05, 95% *CI* = 1.01–1.10).

### Research question 3. the study of vulnerable adolescents

This third section focuses on those adolescents who, despite having good-quality parent-child relationships show low global health scores, that is to say, the vulnerable group (3.9% of the global sample and 11.9% of the group of participants that showed high-quality in parent-child relationships). This group of adolescents are compared with those adolescents who, having a good-quality parent-child relationship, show high global health score, that is to say, the competent group (18.3% of the global sample and 56.1% of the group with good-quality parent-child relationships).

Results from the logistic regression analyses, taking the group of vulnerable adolescents as a reference value, are shown. As in the analyses of the resilient adolescents, six models have been estimated, one for each set of independent variables (including the variables sex and age in all of them, so that they do not become confounding variables). In addition, a global model is presented at the end in which only the significant variables from previous models are included.

As can be seen in the first row of Table 9, although model 1 overall explained 9.7% of total variability, with the variables sex and age being significant (specifically, girls and older adolescents have a higher probability of being vulnerable), the percentage of correctly classified adolescents using theses demographic variables only was 0%.

**Table 9:** **Logistic regression models on vulnerability by school context, peer context, lifestyle, psychological and socioeconomic variables**.

		**Model 1-OR (95%-CI) Demographic variables**	**Model 2-OR (95%-CI) School context**	**Model 3-OR (95%-CI) Peer context**	**Model 4-OR (95%-CI) Lifestyles**	**Model 5-OR (95%-CI) Psychological variables**	**Model 6-OR (95%-CI) Socioeconomic variables**	**Model 7-OR (95%-CI) Global**
	**Variables**	***R*^2^ = 0.097 (81.5/0.0%)**	***R*^2^ = 0.204 (82.3/16.8%)**	***R*^2^ = 0.168 (81.3/10.0%)**	***R*^2^ = 0.210 (82.8/17.5%)**	***R*^2^ = 0.447 (83.3/52.0%)**	***R*^2^ = 0.112 (82.4/2.7%)**	***R*^2^ = 0.565 (86.6/62.9%)**
**Demographic variables**	Sex (ref. girls)	0.46^**^ (0.35–0.60)	0.35^**^ (0.26–0.47)	0.41^**^ (0.31–0.55)	0.62^**^ (0.45–0.85)	0.59 (0.33–1.05)	0.52^**^ (0.36–0.76)	0.76 (0.38–1.53)
	Age	1.60^**^ (1.41–1.80)	1.53^**^ (1.34–1.74)	1.57^**^ (1.38–1.79)	1.42^**^ (1.24–1.62)	1.36 (0.92–2.02)	1.62^**^ (1.37–1.91)	1.31 (0.85–2.01)
	Type of educational center (ref. public)	0.77 (0.58–1.03)	NA	NA	NA	NA	NA	NA
	Habitat (ref. urban)	0.80 (0.61–1.06)	NA	NA	NA	NA	NA	NA
**School context**	Perceived academic achievement	NA	0.60^**^ (0.50–0.72)	NA	NA	NA	NA	0.49^**^ (0.32–0.76)
	Feelings toward school	NA	0.77^**^ (0.65–0.91)	NA	NA	NA	NA	0.88 (0.62–1.27)
	Perceived teacher support	NA	0.87^**^ (0.83–0.91)	NA	NA	NA	NA	0.85^**^ (0.75–0.95)
**Peer context**	Perceived social support	NA	NA	0.97^**^ (0.94–0.99)	NA	NA	NA	1.01 (0.91–1.13)
	Models of behavior	NA	NA	0.98 (0.94–1.02)	NA	NA	NA	NA
	Satisfaction with friendships	NA	NA	0.76^**^ (0.69–0.83)	NA	NA	NA	0.92 (0.74–1.15)
	Having been bullied	NA	NA	1.12 (0.88–1.43)	NA	NA	NA	NA
	Having bullied others	NA	NA	1.10 (0.86–1.41)	NA	NA	NA	NA
**Lifestyles**	Breakfast frequency	NA	NA	NA	0.94 (0.87–1.02)	NA	NA	NA
	Fruit consumption	NA	NA	NA	0.99 (0.91–1.08)	NA	NA	NA
	Snack consumption	NA	NA	NA	1.05 (0.93–1.18)	NA	NA	NA
	MVPA	NA	NA	NA	0.74^**^ (0.67–0.80)	NA	NA	0.70^**^ (0.58–0.86)
	VPA	NA	NA	NA	0.89^*^ (0.80–0.99)	NA	NA	0.84 (0.67–1.05)
	Dental hygiene	NA	NA	NA	0.70^**^ (0.56–0.89)	NA	NA	1.54 (0.79–2.99)
	Tobacco use	NA	NA	NA	1.03 (0.84–1.26)	NA	NA	NA
	Alcohol use	NA	NA	NA	1.45^**^ (1.20–1.76)	NA	NA	1.24 (0.84–1.82)
	Cannabis use	NA	NA	NA	1.19 (0.86–1.65)	NA	NA	NA
**Psychological variables**	Sense of coherence	NA	NA	NA	NA	0.27^**^ (0.18–0.40)	NA	0.30^**^ (0.19–0.45)
	Emotional regulation	NA	NA	NA	NA	0.99 (0.94–1.03)	NA	NA
	Curiosity and exploration	NA	NA	NA	NA	0.95^**^ (0.92–0.98)	NA	0.96^*^ (0.93–0.99)
	Perceived body image	NA	NA	NA	NA	0.76 (0.50–1.16)	NA	NA
	Satisfaction with body image	NA	NA	NA	NA	0.46^**^ (0.35–0.69)	NA	0.50^**^ (0.35–0.71)
**Socioeconomic variables**.	Father educational level	NA	NA	NA	NA	NA	1.19 (0.89–1.58)	NA
	Mother educational level	NA	NA	NA	NA	NA	0.66^**^ (0.51–0.87)	0.83 (0.57–1.23)
	Perceived family wealth	NA	NA	NA	NA	NA	0.65 (0.41–1.02)	NA

MVPA, Moderate to Vigorous Physical Activity; VPA, Vigorous Physical Activity; OR, Odds Ratio (95% CI, Confidence Interval at the 95% level); R^2^ = Model explained variancel (% correctly-classified total/% correctly-classified vulnerable group); NA, not applicable.

* p < 0.05,

** * p < 0.01*.

In model 2, regarding school context, the explained variance is 20.4% and the model correctly classifies 16.8% of the vulnerable adolescents. Specifically, those adolescents who perceive lower teacher support, with an OR of 0.87 (95% *CI* = 0.83–0.91), have less positive feelings toward school (*OR* = 0.77, 95% *CI* = 0.65–0.91) and worse academic achievement (*OR* = 0.60, 95% *CI* = 0.50–0.72) have a higher likelihood of being vulnerable.

Model 3, which includes the variables of peer context, shows a predictive capacity of 16.8%, with 10% of the vulnerable adolescents being correctly classified. Those adolescents who report lower perceived social support (*OR* = 0.97, 95% *CI* = 0.94–0.99) and less satisfaction with friendships (*OR* = 0.76, 95% *CI* = 0.69–0.83) are more likely to be vulnerable.

Model 4 is devoted to variables related to lifestyles and its explained variance level is 21%, with 17.5% of the vulnerable adolescents being correctly classified. In this model, alcohol use stands out, showing that those adolescents who show a higher frequency of alcohol use in the last 30 days are 1.45 times more likely to be vulnerable (95% *CI* = 1.20–1.76). Likewise, the adolescents who do less moderate to vigorous physical activity (*OR* = 0.74, 95% *CI* = 0.67–0.80) and less vigorous physical activity (*OR* = 0.89, 95% *CI* = 0.80–0.99) have a higher likelihood of being vulnerable.

The group of psychological variables, analyzed in model 5, has the highest level of explained variance among the six specific models. Specifically, the level of explained variance in model 5 is 44.7% and 52% of the vulnerable adolescents are correctly classified. As in the previous section regarding resilient adolescents, the significant variables in this model are: sense of coherence, curiosity and exploration and satisfaction with body image. The likelihood of being vulnerable is higher in those adolescents with a lower score in sense of coherence (*OR* = 0.27, 95% *CI* = 0.18–0.40), less satisfaction with their body image (*OR* = 0.46, 95% *CI* = 0.35–0.69) and a lower score in curiosity and exploration (*OR* = 0.95, 95% *CI* = 0.92–0.98).

Model 6, examining the socioeconomic variables, again shows a lower predictive capacity than previous models (11.2%), with only 2.7% of the vulnerable adolescents being correctly-classified. The mother's educational level is the only significant variable in this model, revealing that those adolescents whose mothers have a lower educational level exhibit a higher probability of being vulnerable (*OR* = 0.66, 95% *CI* = 0.51-0.87).

Lastly, in model 7 or the global model (in which only the significant variables from previous models have been included), the following six variables were significant: perceived academic achievement, perceived teacher support, moderate to vigorous physical activity, sense of coherence, curiosity and exploration and satisfaction with body image. The predictive capacity of this model is very high, with an explained variance level of 56.5%. This model was also able to correctly classify a high proportion of vulnerable adolescents, specifically 62.9%.

The independent variables which stand out in this model due to their ORs being closer to zero, and therefore their higher predictive capacity, are sense of coherence (*OR* = 0.30, 95% *CI* = 0.19-0.45), academic achievement (*OR* = 0.49, 95% *CI* = 0.32–0.76) and satisfaction with body image (*OR* = 0.50, 95% *CI* = 0.35–0.71). Moderate to vigorous physical activity (*OR* = 0.70, 95% *CI* = 0.58–0.86) and teacher support (*OR* = 0.85, 95% *CI* = 0.75–0.95) appear on an intermediate level. Lastly, the level of curiosity and exploration (*OR* = 0.96, 95% *CI* = 0.93–0.99) made the most modest contribution. Higher levels of the aforementioned variables are associated with a lower likelihood of belonging to the group of vulnerable adolescents.

## Discussion

The aim of this study was to characterize resilience and vulnerability in a large and representative sample of adolescents. This objective was first addressed separately on a number of potential levels of influence (demographic, school, peer, lifestyle, psychological, and socioeconomic variables) and later, in a more holistic approach, by integrating the factors in all the aforementioned levels.

A separate analysis of each of the two phenomena showed, first at all, that although there was a higher representation of boys and younger adolescents in the resilient group, and of girls and older adolescents in the vulnerable group, the variables sex and age were not sufficient to accurately predict adolescent adaptation. Previous research has found differences in wellbeing and adjustment between boys and girls, as well as according to age (Cavallo et al., 2006; Ramos et al., 2010), but at the same time there is notable diversity amongst adolescents of the same sex and age. This diversity tends to be related to the combination of life experiences and psychological characteristics of these adolescents. Hence the demographic variables (that were included in all the regression models) were insufficient to characterize such complex phenomena such as resilience and vulnerability and their significant effects disappeared when they were entered along with the rest of the variables in the final model. In fact, sex and age already lost their significant effects in previous models, specifically in those evaluating the contributions of psychological and socioeconomic variables. This is probably owing to that those models incorporated variables such as satisfaction with body image, which tends to be lower and more strongly associated with girls' wellbeing (Knauss et al., 2007; Mond et al., 2011), or family wealth, which tends to be assessed more negatively by older adolescents (Goodman et al., 2001). Therefore, it could be understood that these predictor variables (such as body image or family wealth) explain the predictive capacity of the variables sex and age on the phenomena resilience and vulnerability.

Beyond demographic variables, a look to the separate models for each set of predictors shows that a hierarchy based on the predictive capacity of each set of variables would be very similar for resilience and vulnerability: psychological variables in the first place, along with contextual and lifestyle variables, and more modest contributions of demographic and socioeconomic variables.

In addition, the final models for resilience and vulnerability also revealed a number of common factors for the explanation of both phenomena. In other words, these analyses also helped identify several factors that contributed significantly to explaining both resilience and vulnerability.

First at all, sense of coherence was one of the most important factors for both resilience and vulnerability. This construct, coming from the salutogenic model in the field of public health, has to do with a person's ability to interpret their social environments as predictable and ordered, their confidence that any life demand can be successfully dealt with as well as a motivational-emotional component that helps one to see difficult situations as challenges and facilitates an active engagement in problem-solving (Antonovsky, 1987). Therefore, the important contribution of sense of coherence to resilience and vulnerability should come as no surprise. On one hand, its links to some factors associated with successful adaptation in classic resilience studies, such as analytical skills, motivation to engage in the environment, self-efficacy and self-esteem (Masten, 2001; Hamill, 2003), are apparent in the prior description. In addition, research on sense of coherence indicates that its relationship with health and wellbeing is rooted in helping people mobilize other useful coping resources in stressful situations (Lindström and Eriksson, 2010), which has led to its inclusion in the health assets model as a *supra-order asset* for wellbeing (Morgan and Hernán, 2013). In this sense, one line of research that arises from the results obtained in the current study is the study on the processes that explain why a high sense of coherence would help resilient adolescents take full advantage of available resources, whereas low levels of the same would hamper the effective use of the apparently more abundant resources in the case of vulnerable adolescents.

Satisfaction with body image and perceived academic achievement also appeared as important explanatory variables in the analysis of both resilience and vulnerability. The significant contribution of satisfaction with body image is probably related to the importance of physical appearance for adolescents' positive self-perception. In this regard, numerous studies have found a significant relation between satisfaction with body image and self-esteem in adolescence (Tiggemann, 2005), this latter being a factor traditionally connected to successful adaptation (e.g., Dumont and Provost, 1999). Something similar can be said of the relationship between perceived academic achievement and self-efficacy (Danielsen et al., 2009), another fundamental protective factor in resilience research (Hamill, 2003). Additionally, previous research indicates that feeling competent in daily life is very important for the adaptation of individuals suffering adversity (Masten and Coatsworth, 1998). Therefore, it is likely that behind an adolescent who thinks that their teachers consider their academic achievement as good, there are various underlying beneficial elements for adaptation and wellbeing, such as experiences of competence in the school context, higher school connectedness or even a higher intellectual capacity (Masten et al., 1999; Blum, 2005).

In addition to perceived academic achievement, higher levels of teacher support increased the likelihood of showing resilience and diminished that of being part of the group of vulnerable adolescents. Studies about teachers' contribution to adolescent wellbeing also suggest that, regardless of the level of academic achievement, teacher support acts as an asset associated with wellbeing for all adolescents (e.g., García-Moya et al., 2015), which makes it fundamental to favor close and supportive teacher-student relationships.

Moderate to vigorous physical activity was also amongst the significant factors associated with resilience and vulnerability. Physical activity has been found to have protective effects in stressful situations (Gerber and Pühse, 2009; Silverman and Deuster, 2014), as well as it tends to reduce the likelihood of engaging in risk behaviors (Pate et al., 1996), therefore serving as a clear example of the importance of taking into account lifestyles' contributions to explaining resilience and vulnerability.

Finally, higher levels of curiosity and exploration increased the likelihood of being resilient and diminished that of being part of the vulnerable group. The curiosity and exploration construct reflects openness and interest in learning, good management of the uncertainty associated with new or unknown situations (Kashdan et al., 2009) and is associated with psychological and contextual variables significantly linked to adaptation and resilience. Specifically, high levels of curiosity and exploration are related to an active response in unfamiliar and challenging environments (Kashdan and Roberts, 2004) and have been linked to psychological variables such as intrinsic motivation and self-efficacy (Kashdan et al., 2004). Additionally, curiosity was also significantly associated with more positive social interactions (Kashdan and Roberts, 2004). Specifically, people with higher levels of curiosity and exploration generated more positive responses from strangers, who tended to be more responsive, participative, and interested in social exchanges with people with high curiosity. Despite this, the contribution of curiosity and exploration to the final model was relatively modest, probably due to its connections with other constructs, such as sense of coherence. The conceptual delimitation of curiosity and exploration is still under study (Kashdan et al., 2009), and with regards to sense of coherence one focus of analysis and debate is precisely its connection to other constructs in positive psychology (Lindström and Eriksson, 2010). Consequently, advancing in the conceptual delimitation of these constructs, identifying common elements and differences between them, is an important line of research (García-Moya and Morgan, 2016) that could contribute to a better understanding of resilience and vulnerability and, in general, of their role in promoting adolescent wellbeing and adjustment.

As explained in the previous lines, the vast majority of the examined factors operated by increasing the likelihood of good adaptation in resilient adolescents and diminishing it in vulnerable ones. Overall, this suggests more similarities than differences in the factors contributing to explaining resilience and vulnerability. These findings coincide with previous research pointing out that factors associated with resilience are not specific to this phenomenon, but that they are the manifestation of basic systems of human adaptation and, therefore, are influential in both adversity and non-adversity situations (Masten and Coatsworth, 1998; Masten, 2001). Additionally, some scholars have noted that protective factors identified in resilience and vulnerability studies frequently correspond to the positive pole of risk factors for maladaptation or, in other words, that in this type of research it is possible to identify factors in which one of their extremes facilitates successful adaptation while the opposite hampers it (Sameroff and Fiese, 2000; Fergus and Zimmerman, 2005; Luthar et al., 2015), which seems to coincide with findings in the present study.

Despite the predominant similarities described so far, results also revealed some differential aspects between the resilience and vulnerability phenomena. First, the psychological variables showed a larger explicative capacity in vulnerable adolescents than in resilient ones (*R*^2^ = 0.447 and 0.370, respectively), whereas factors related to school and peer contexts, especially the second, showed a stronger association with resilience than with vulnerability (*R*^2^ = 0.228 and 0.238 respectively in the models on resilience vs. 0.204 and 0.168 for vulnerability). Some research suggests that certain protective factors such as temperament (e.g., Werner and Smith, 1982) or intellectual capacity (Masten and Coatsworth, 1998), to name some classic examples, have a multiplier effect, i.e., they can contribute to a higher likelihood of encountering other positive events in life, giving rise to chain reactions favoring positive adaptation or, conversely, they can initiate cascading effects in which new risk factors are more probable. Applying a similar logic, it can be hypothesized that certain psychological variables, such as a low sense of coherence, a lower tendency toward curiosity and exploration, or a higher dissatisfaction with body image, could be preventing vulnerable adolescents from taking advantage of potential resources in extrafamily environments (school and peer contexts), whereas resilient adolescents, despite their more unfavorable family context (which was the indicator used for the definition of adversity in the current study), would be more likely to find and benefit from resources available in extrafamily environments thanks to their more positive profile in psychological variables. Along these lines, prior research has documented the existence of compensatory effects from other contexts in only part of the adolescents exposed to low-quality family contexts (e.g., García-Moya et al., 2013b).

Second, three of the examined factors, specifically perceived family wealth, satisfaction with friendships and breakfast frequency, were only significant in the analysis of resilience. This means that these variables made a difference for adolescents exposed to adversity in the family context (resilient vs. maladaptative adolescents) but did not contribute to explain differences in adaptation between vulnerable and competent adolescents. Scientific literature has extensively documented that resilience has among its defining attributes an ability, despite adversity, to find and take advantage of any resources and opportunities in proximal environments.

In this sense, it is not surprising that being raised in a family environment with good socioeconomic resources opens a horizon of possibilities to resilient adolescents that they seem to know how to take advantage of. What is interesting in the findings of the present study is that although vulnerable and resilience adolescents reported similar levels of perceived family wealth, this factor made one of the most noticeable contributions in analyzing resilience (but not vulnerability). A reflection on the nature of the indicator used may help understand this finding. On the one hand, research suggests that perceived family wealth includes some of the elements which are common to objective indicators such as family affluence, and therefore, it can arguably be interpreted as indicative to some extent of the wider access to external resources and opportunities for development that families' socioeconomic level relates to Bornstein and Bradley (2003). However, research also indicates that subjective and objective measures are not assessing exactly the same content (Hartley et al., 2015; Elgar et al., 2016), since unlike objective indicators perceived family wealth may also incorporate a comparative assessment of the socioeconomic position of the adolescent's family in comparisons with that of others they related with (Moreno-Maldonado et al., under review). The levels of wealth perceived by resilient adolescents may therefore represent a relative socioeconomic advantage for these adolescents compared to their peers also exposed to adversity in family relationships (the maladaptative group).

Results on satisfaction with friendships can also be interpreted in a similar sense, i.e., that resilient individuals are able to take advantage of the potential resources they find. Peer support tends to be considered a protective factor in adversity situations (Olsson et al., 2003) and resilient adolescents in the present study probably illustrate very well the compensatory effects which are frequently mentioned in this field (e.g., Luthar et al., 2015): they belong to a group who, despite coming from families in which parent-child relationships are not good, is able to build positive relationships with their peer group and benefit from them (Lansford et al., 2003; Rubin et al., 2004). In a similar vein, Luthar et al. (2015) state that relationships with peers can become a “remedial” socializing context for children who grow up exposed to family adversity. In addition, positive peer relationships are indicative of good social competence, a fundamental skill in which resilient adolescents usually show positive results, which are comparable to those of competent adolescents and clearly more favorable than the social competence levels exhibited by maladaptative adolescents (Masten et al., 1999).

Finally, the fact that breakfast frequency was significant only in the analysis of resilience may be related to the fact that, as children gain more independence during adolescence, the importance of parental supervision in this behavior decreases while internalization of the habit and other personal characteristics, such as constancy and self-regulation, gain prominence (Kalavana et al., 2010). Given that breakfast frequency is also believed to act as a proxy for diverse socioeconomic and family aspects this is an issue which, in particular, would benefit from further research.

In any case, the comments that have been made throughout this discussion about a higher ability of resilient individuals to take advantage of potential resources in proximal contexts or the important role of psychological factors for explaining the resilience and vulnerability phenomena should not be interpreted as evidence that they are characteristics unrelated to the contextual experiences associated with resilience and vulnerability. As rightly pointed out by Luthar et al. (2015), contextual experiences indeed give shape, from the beginning and in a continuous transactional dynamic, to said skills or psychological resources.

This study has some limitations that should be taken into consideration in the interpretation of its findings. Firstly, its cross-sectional design means that the results must be interpreted on an associative level, not being possible to draw conclusions about the directionality of the relationships found. Secondly, adversity was defined using quality of parent-child relationships as a sole criterion. Although, as explained in the introduction, this is an well-informed decision, which draws on scientific literature that highlights the role of family as a basic system for human adaptation (Masten, 2001; Fergus and Zimmerman, 2005), previous research also shows the wide variety of life circumstances that can constitute adversity in childhood and adolescence (Luthar et al., 2000); consequently, it would be inappropriate to generalize these findings to other adverse circumstances. Finally, this study used a factorial health score as its measure of adaptation. Although, this measure is a sound and validated global health indicator (Ramos et al., 2010) whose characteristics fit well with key measurement issues in the empirical definition of positive adaptation (Luthar and Cushing, 1999), there is substantial evidence on the multi-dimensional nature of human adaptation, which makes individuals show dissimilar levels of adaptation in different areas (Luthar et al., 1993). Therefore, future research should complement the present study by conducting separated analyses of the contributions of the factors analyzed here to distinct areas of adaptation, mainly the following: academic, behavioral, social and emotional (Masten et al., 1999; Luthar et al., 2000).

Despite the aforementioned limitations, this study also has important strengths. In line with recommendations from some of the seminal reviews in this research field (Luthar et al., 2000, 2015; Masten, 2014), the elements of adversity and adaptation were clearly operationalized for the definition of resilience and vulnerability in the present study, which is fundamental for an adequate interpretation of its findings and its comparability with other studies. The criteria used for making the distinction and comparisons among the four adaptation groups (competent, vulnerable, resilient, and maladaptative) were also based in previous research (Tiêt and Huizinga, 2002). Additionally, this research adheres to the methodological rigor characteristics of the HBSC survey (Roberts et al., 2009), as well as it stands out for its large sample size, which allowed for a characterization of resilience and vulnerability phenomena in a representative and notably large sample. Although, the four groups may appear unbalanced in size, the representativeness of the initial sample is a guarantee that this is a relatively realistic reflection of the population. In addition, using effect size tests in all of the analyses minimizes the potential bias that such differences in the subgroups' size could case from a methodological point of view. The high predictive capacity of the models of resilience and vulnerability obtained, which reached levels of explained variance higher than 50%, is also outstanding. These values are considered high in the field of behavioral science (Cohen, 1988), being notably above the 10–20% that is usual for associations between protective factors and adaptation outcomes in resilience studies (Luthar et al., 2000). Finally, this study has three elements that are, to some extent, innovative. Firstly, factors traditionally receiving little attention as referred to in the introduction, such as lifestyles, satisfaction with body image, sense of coherence, curiosity and exploration and perceived family wealth, were analyzed in the present study of resilience and vulnerability. Secondly, this study included vulnerable adolescents, a population subgroup that had rarely been studied in previous research due to its limited sample size (Masten et al., 1999). Additionally, this work makes a valuable contribution regarding the prevalence of vulnerability and resilience in the general population. Given the difficulties associated with defining resilience and vulnerability and the limited methodological consensus with regards to the measures to use and how to apply them to a representative sample, it is understandable that prevalence studies are not available. In this regard, the present study found that vulnerable adolescents made up 3.9% of the global sample, representing 11.9% of the group that reported high-quality parent-child relationships. The resilience group represented 4.5% of the global sample, corresponding to 13.4% of the participants with low-quality parent-child relationships, which is in line with the findings of some longitudinal studies that have found a very low prevalence and stability in resilient coping (Cicchetti and Rogosch, 1997). Specifically, Bolger and Patterson (2003) found that between 6 and 21% of abused children were functioning competently during at least one of the temporal points examined in their longitudinal follow up from middle childhood to early adolescence, but less than 5% consistently maintained that competent functioning over time.

In addition to its strengths from a research perspective, which have just been highlighted, the fact that the present study provides valuable implications for the improvement of the methodological quality of interventions with resilient and vulnerable populations, which was one its guiding principles, should also be noted amongst its strong points.

Throughout these pages a number of important factors for adolescents' successful adaptation have been underlined. These include certain personal characteristics (such as sense of coherence, satisfaction with body image and curiosity and exploration), as well as some that characterize their lifestyles (regularity in healthy eating habits and physical activity) or that refer to the quality of their developmental contexts (such as satisfaction with peer relationships, academic achievement and teacher support). Therefore, all of these are elements to bear in mind in interventions aimed at promoting successful adaptation and wellbeing in adolescence (Olsson et al., 2003). Likewise, this study highlights the need to conduct further research devoted to developing reliable and valid indicators for the assessment of all these factors, both those that characterize the individual person and the ones that characterize their developmental contexts. These indicators will serve the double function of detecting subjects with different profiles of adaptation as well as of monitoring their evolution and evaluating the implemented interventions.

On a separate issue, it should be noted that some studies have advocated that interventions should be adjusted to the distinct developmental needs of adolescence (Kim et al., 2015). What the present research adds is that detecting different adaptation profiles would also serve to adjust interventions to every person's specific needs. On the one hand, some could argue that allocating powerful and costly resources to detect and intervene in vulnerable individuals, which represents 3.9% of adolescents, would not be an efficient strategy. However, it must be noted that this study used very demanding criteria to define the categories of vulnerability/resilience, and hence may have underestimated the prevalence figures. Additionally, it is well known from the accumulated evidence in previous research that life paths of vulnerable people will be full of difficulties in very different areas (this paper has provided some good examples of this). From an economic perspective, those adverse life paths will lead to a lot of public spending in the education, health, legal and judicial, and labor systems, amongst others (see Khan et al., 2015), if the direct and indirect costs to which these difficulties will give rise are taken into consideration; consequently, they should be detected as soon as possible. On the other hand, one should not forget that amongst the adaptation profiles considered in this paper, there were also a 18.88% of maladaptative adolescents with clear intervention needs, and in the remaining 77.3% of adolescents there will most likely always be areas of improvement and optimization in need of reinforcement. Similarly, it could also be thought that the resilient adolescents, for which our study shows a prevalence only slightly higher than 4%, would not need any intervention because they seem to resist adversity without help. It is true that these adolescents seem to have an admirable capacity to deal with adversity, but their resilience is not without limits. As can be seen when comparing them to the competent adolescents (please compare values of the resilient column with values of the competent column in Tables 4, 5), resilient adolescents scored lower in an important number of variables. In other words, even though adolescents in the resilient group showed very high levels of adjustment despite coming from adverse family environments, their adjustment levels could still be higher if they were aided in taking full advantage of their skills and if interventions were implemented at the source of adversity. Needless to say, reducing adversity in their family environment should also be a top priority. Additionally, certain studies have already warned on the risks of underestimating resilient adolescents' needs for support, since some of these adolescents, despite being classified as resilient for showing excellent competence levels according to external and behavioral indicators, can nonetheless suffer from elevated levels of emotional distress (Luthar et al., 1993).

A final more general consideration should probably be added. In the dynamic relationship between research and intervention underlined in this paper, it should be emphasized that interventions should not work with models that explain development and change from a lineal or even interactive perspective, since empirical evidence shows data in favor of transactional models that involve much more complex multilevel dynamic systems (Sameroff, 2010). Therefore, even though all recent school intervention efforts aimed at strengthening life skills to optimize development and along the way prevent risk behaviors deserve our most sincere recognition and applause (Springer et al., 2004), the intervention that we defend here should go further. This guiding conceptual framework leads to the claim that intervention in adolescence should be preceded by an ambitious systematic and multi-sector intervention starting at the beginning of life. In this vein, as already noted by Luthar and Cicchetti (2000), interventions should take into consideration and simultaneously work on different levels of influence (individual, family and extrafamily) and should begin as early as possible. Community work with families and current steps toward promoting positive parenting very early in the baby's life are good points of reference in this direction (Rodrigo et al., 2012).

In summary, this study emphasizes the enormous potential of research on resilient and vulnerable individuals, both for creating scientific knowledge and for designing intervention guidelines. For a long time psychology overlooked both phenomena (vulnerability and resilience), due to the predominant scientific interest in central trends, i.e., toward what happened to the majority of people. Research was focused, on one hand, on those individuals that succumbed to adversity, and on the other, on those that showed strength as the result of having grown up surrounded by quality relationships. However, psychology must acknowledge the great deal that has been learnt since then by studying the limited percentage of people whose developmental trajectories apparently challenged the central-tendency hypotheses of that time: individuals who appeared to be strong and healthy despite adversity, as well as those who, despite growing up surrounded by strengths, seemed to be weak. Analysing the life trajectories of the first helps us to clarify what is desirable that all people have in their lives and the analysis of the life paths of the second, teaches us what is necessary to eradicate in all of them.

## Ethics statement

The study was approved by the ethics committee of Comité Ético de Experimentación de la Universidad de Sevilla. Parents of adolescents participating in the study received a letter with information about the study and informed consent model.

## Author contributions

All authors conceived of the study, participated in its design and helped to draft the manuscript. Introduction was drafted by IG, methods and results were drafted by PR, FR and the discussion draft was done by IG, CM. All authors made suggestions and critical reviews to the initial draft and contributed to its improvement until reaching the final manuscript, which was read and approved by all authors.

### Conflict of interest statement

The authors declare that the research was conducted in the absence of any commercial or financial relationships that could be construed as a potential conflict of interest.
